# Prevalence and correlates of sexual behaviors among university students: a study in Hefei, China

**DOI:** 10.1186/1471-2458-12-972

**Published:** 2012-11-13

**Authors:** Xinli Chi, Lu Yu, Sam Winter

**Affiliations:** 1 Department of Education, University of Hong Kong, Room 101, HOC BLOG, Hong Kong, China; 2 Department of Applied Social Sciences, Hong Kong Polytechnic University, Hong Kong, China

## Abstract

**Background:**

In China, sexual health and behaviors of young people have become a growing public concern but few studies have been conducted to investigate the prevalence and psychosocial correlates of the phenomenon.

**Methods:**

A self-reported questionnaire survey on youth sexual behaviors was conducted among 1,500 university students in 2011 at Hefei, a middle-size city in eastern China. A total of 1,403 students (age = 20.30 ± 1.27 years) completed the questionnaire with a high response rate of 93.5%.

**Results:**

Among the respondents, 12.6% (15.4% of male versus 8.6% of female) students reported having pre-marital heterosexual intercourse; 10.8% (10.5% of males versus 11.2% females) had oral sex; 2.7% (3.4% of males versus 1.7% females) reported same-sex activities; 46% (70.3% of males versus 10.8% of females) reported masturbation behaviors; 57.4% (86.2% of males versus 15.6% females) students viewed pornography. In terms of sexual communication about sexual knowledge acquisition, 13.7% (10.7% of males versus 18% of females) talked to their parents about sex; 7.1% (6.1% of males versus 8.4% of females) students reported having conversation with parents on contraception. About forcing sexual behavior, 2.7% (4% of males versus 0.9% of females) reported forcing their sexual partners to have sex, and 1.9% (2.4% of males versus 1.2% of females) reported being forced to have sex. Gender was found to be significant predictor of sexual behaviors in university students: males reported more sexual behaviors including sexual fantasy, heterosexual intercourse, masturbation, viewing pornography and talking about sex with friends. Several correlates of sexual behaviors were identified for students of different gender separately. For males, having romantic relationships, past sex education experiences, low educational aspirations, time spent on the Internet, and urban native settings were significantly associated with more sexual behaviors. For female students, having romantic relationships and urban native settings predicted sexual behaviors.

**Conclusion:**

Sexual behavior among University students in China is not uncommon, although there are limited ways for students to acquire sex-related knowledge: male students showed significantly more sexual behaviors than female students. Having romantic relationships and more time spent online were important predictors of sexual behaviors among university students. To guide healthy sexual behaviors in young people, comprehensive sex education programs that provide necessary sexual health knowledge about safe sex should be developed and implemented in universities in China, particularly for students who have romantic relationships and those who spend long periods of time on the Internet.

## Background

Young people are at the beginning of their sexual and reproductive lives. How they are prepared for this journey has tremendous implications for their future lives and the health of the next generation. Sexual behaviors refer to a variety of sexual acts, such as talking about sex, solitary masturbation, intimacy, and sexual intercourse through their experiences and expressing their sexuality. Youth sexual behavior is highly relevant to different public health problems [1,2]. For example, youth unprotected sexual intercourse contributes to unwanted pregnancies, abortions, pregnancy related complications, and sexually transmitted infections (STIs) including HIV/AIDS [3]. While there are numerous studies on sexual behaviors and health among young people in Western countries, such investigations have been rare in different Chinese communities. Understanding the prevalence and psychosocial correlates of sexual behaviors in young Chinese people would provide important information on the development and implementation of effective sex education programs in China and thus help Chinese youth develop healthy and safe sexual behaviors. As such, the present study aims to investigate the prevalence and psychosocial correlates of sexual behaviors in young Chinese people based on a large sample of University students in Hefei, a typical middle-sized city in China.

In recent decades, a large number of studies have examined the prevalence of youth sexual behaviors usually in multiple aspects of heterosexual and homosexual intercourse, sexual coercion, masturbation, and pornography viewing in Western countries [4]. For example, a study found 80% of the males and 73% of the females had experienced heterosexual intercourse in United States [5]. Overall, 74% of university students reported ever having had sexual intercourse in Turkey [6]. A report conducted among 8658 American university students found 5% students had sexual experiences with members of their own sex [7]. Worldwide evidence shows that the experience of sexual coercion is fairly prevalent among young people. Approximately 25–33% of college women in the United States reported experiencing forced touching of sexual parts and around 10% of college women reported experiencing forced oral, anal, and/or vaginal intercourse [8]. It was found that 92% of men and 77% of women university students had masturbated in the United States [9]. In Denmark 97.8% of males and 79.5% of females watched pornography among 1002 people aged from 18–30 years old [10].

In China, a limited number of studies have attempted to examine the occurrence of sexual behaviors in university students in the past two decades. In 1989, a general survey on university students’ lives was conducted in Beijing, which revealed that around 13% of male students and 6% of female students had encountered sexual experiences [11]. In 1992, a similar study in Shanghai indicated that 18.8% of male university students and 16.8% of female students had engaged in premarital sex [12]. In 2000, a nationwide survey involving over 5000 participants from 26 universities in 14 provinces revealed that 11.3% of university students experienced sexual intercourse [13]. Meanwhile, a multi-year longitudinal study in Beijing revealed that the percentage of premarital sex among university students rose from 16.9% in 2001 to 32% in 2006 [14]. While these results provided useful information about the general situation of sexual behaviors in Chinese university students, most studies only adopted single-item “Yes” or “No” questions to obtain information regarding whether the student had sexual intercourse or not. Multiple aspects of sexual behaviors, such as same-sex sexual contact and sexual communication, are unknown. Obviously, such studies cannot provide a clear picture about Chinese university students’ sexual behaviors in different aspects. Therefore, there is a critical need to further investigate this phenomenon by using comprehensive assessment tools which can assess multiple dimensions of youth sexual behaviors in China.

Several important predictors of youth sexual behaviors have been reported by Western researchers. A study revealed students with lower grades, compared to those with higher grades, were more likely to have sexual intercourse experience in high school [15]. College students from the cities and towns were more prone to sexual intercourse than those from a rural area [16]. In high school, teenagers with bad school achievements were more likely to have already lost their virginity and engage in more sexual activities than those who achieved academic success [17]. In terms of sex education, it has been a controversial subject in some countries for a long time, such as the USA and China. A large number of studies conducted across the world examined it and its association with youth’s sexual behaviors. Some found sex education may reduce the level of sexual activities and risk sexual behaviors [18]. Some findings found that sex education might not cause any sexual behavior changes [19]. Studies commonly demonstrated that among adolescents and young people, being in a romantic relationship is significantly associated with an increased likelihood of sexual initiation and sexual activities [20]. Also, exposure to the Internet and the messages they present are greatly influential factors on American adolescents [21]. Although studies have yielded valuable information to understand psychosocial correlates of sexual behaviors among adolescent and young people, most of such studies were conducted with western contexts. There is no research examining whether and how these factors also affect Chinese university students’ sexual behaviors. The present study was the first study to attempt to explore psychosocial correlates of sexual behaviors based on Chinese context.

Furthermore, factors that affect sexual behaviors appear to be different for males and females. Many studies in western countries indicate that males are more likely to initiate sexual intercourse, higher prevalence rate, more frequent sexual behaviors and significantly higher risk behavior than girls [22,23]. Males were more likely to report having lost their virginity and having initiated sexual intercourse in earlier age and more sexual partners than females [24]. Young people often learn from experience, and their experiences may influence their subsequent behavior. This process may vary for male and female youth, since society often places different meanings on sexual activity for males and females. For example, stronger social and emotional sanctions have been associated with sexual activity for females than for males [25]. Males tend to receive more permissiveness from society for premarital sexual activity than females. Given that sexual behaviors have different implications for males and females, psychosocial factors associated with sexual behaviors will vary as well. However, it is unclear how gender differences on prevalence of sexual behaviors are; what and how psychosocial factors linked to sexual behaviors for Chinese male and female students. Thus, it is necessary to explore how gender differences on prevalence of sexual behaviors; what and how psychosocial factors were associated with sexual behaviors for males and females, respectively.

Against the research background, the present study was designed to address three primary research questions: (a) how prevalent are sexual behaviors among university students in Hefei. (b) What are the gender differences in prevalence of sexual behaviors? (c) What are the factors associated with sexual behaviors for males and females, respectively?

## Methods

### Procedure and participants

The present study was conducted in Hefei, a typical middle-size city in Eastern China in September, 2010. There are nine comprehensive public universities in Hefei, covering a wide range of disciplines such as science, education, law and literature. From the nine universities, four universities were randomly selected, among which 16 classes was selected from four different grades purposely based on the criterion that there are similar numbers of male and female students in the class (the ratio of male to female ranging from 1:1.5 to 1.5:1). Specifically, at each grade in each university, one class was sampled. All students in the selected classes (n = 1,500) were invited to participate in this study and all of them agreed to be participants by signing up a consent form before the questionnaire survey. Of the 1,500 respondents, 1403 students returned completed questionnaires, indicating a high response rate of 93.5%. Student ages ranged from 18 to 25 years (M = 20.30, SD = 1.27), with 59.2% being males and 40.8% being females. Detailed demographic characteristics of the participants are summarized in Table 1.

**Table 1:** Profile of social-demographic characteristics of the sampled participants (n/%)

**Gender**	**Male**		**Female**	
	**n**	**%**	**n**	**%**
	831	59.2	572	40.8
**Age**				
18~19	278	33.5	223	39.0
20~21	361	23.1	266	46.5
22~25	192	32.1	83	14.5
**Grade**				
Year 1	300	36.1	200	35.0
Year 2	179	21.5	160	28.0
Year 3	132	15.9	127	22.2
Year 4	220	26.5	85	14.9
**Discipline**				
Science	630	75.8	328	57.3
Arts	201	24.2	244	42.7
**Education aspiration**				
Bachelor	150	18.1	76	13.3
Master	430	51.7	352	61.5
PhD	251	30.2	144	25.2
**Romantic relationship**				
No	421	50.7	248	43.4
Yes	410	49.3	324	56.6
**Sex education experience**				
No	717	86.3	522	91.3
Yes	114	13.7	50	8.7
**Time spent online**				
Almost no	140	16.8	194	33.9
≤ 2hours	395	47.5	299	52.3
2~4 hours	209	25.2	72	12.6
≥ 4hours	87	10.5	7	1.2
**Rural/Urban area**				
Rural	592	71.2	330	57.7
Urban	239	28.8	242	42.3

The questionnaire survey was conducted by the first author and a trained research assistant in classroom settings with standardized instructions. At each measurement occasion, the purposes of the study were introduced and confidentiality of the data collected was repeatedly ensured to all participants. To maximize the validity of these self-report data, several measures were performed. First, spacious classrooms were used for the survey and students were arranged to sit separately. Specifically, at each survey occasion, a 100-seat lecture hall was provided for no more than 40 students to complete the questionnaire. Second, the first author and the research assistant were present throughout the administration process to answer possible questions. No teachers of the classes or the universities showed up throughout the survey. Third, students were required to focus on their own questionnaires and not allowed to discuss with other students. Fourth, students were encouraged to answer the questions in an honest way and assured repeatedly that their results would be analyzed in an aggregated manner with personal information being kept in strict confidentiality.

This study and data collection procedure has obtained approvals from the surveyed Universities administration committee and human research ethics committee at the University of Hong Kong.

### Measures

#### Socio-demographic characteristics

The first section of the questionnaire consists of questions about the participants’ gender, age, grade (Year 1 to Year 4), study discipline (Science or Art), educational aspiration, romantic relationship experience, sex education experience and the amount of time spent online and area. For educational aspiration, students were asked to indicate which degree they want to achieve in terms of bachelor’s degree, master’s degree, and PhD degree. Two “yes” or “no” questions ask students whether they have any romantic relationships now or in the past; and whether they received sex education before or currently (sex education referred to formal or informal education including courses, workshops, seminars). For time spent online, students were required to report the average number of hours per day they spent on the Internet. One question asked students where they grew up, whether it was in an urban or rural area.

#### Sexual behavior measure

In this study 20 multiple-choice items from Sexual Behavior Inventory of SKAT were used to investigate Chinese university students’ sexual activities *in the last year.* Sexual behavior Inventory was developed by Lief in 1990 [26] and revised by Fullard, Scheier, & Lief in 2005 [27], which is a developmentally appropriate, paper-and-pencil self-report questionnaire for obtaining information about a wide variety of sexual and experiential behaviors relevant to adolescent sexuality and education. The 20 multiple-choice item-questionnaire intended to elicit information about a variety of sexual acts that included kissing, fondling, sexual communication (e.g., *talking with your boyfriend/girlfriend about sex*), intercourse (e.g., *sexual intercourse with a person of the opposite sex)*, and forcing sex. Respondents answered on a Likert scale (*1 = Never, 2 = Less than monthly, 3 = monthly, 4 = weekly, 5 = daily*). Higher total scores indicated having more sexual activities. In order to fit with a Chinese context, the scale was translated and back-translated firstly by two expert bilingual (English and Chinese) (one male and one female) speakers, and then was revised in 14 university students focus group interviews and by 5 expert reviewers, and finally the pilot test with 400 university students samples was conducted in order to gain support for reliability and validity. Finally, 2 items (“*Going home with a stranger you have met at a party or bar” and “Go on a date with a group of friends*”) were irrelevant to Chinese context were deleted and the uni-dimensional Chinese sexual behavior scale with 18 items was developed. The internal consistency of the questionnaire in this study was Cronbach’s alpha = 0.84.

### Statistical analysis

First, frequencies and percentages for each item of the sexual behavior questionnaire were computed to provide a descriptive profile on the prevalence of sexual behaviors among Chinese university students. Second, individual sexual behaviors were compared between male students and female students by independent t-tests to examine gender differences. Thirdly, regression analyses were performed to identify factors that contributed to the occurrences of sexual behaviors, in which participants’ total score on the sexual behavior questionnaire served as dependent variable; age, grade, educational aspiration, romantic relationship experience, sexual education experience, original place/area, and time spent online served as independent variables with gender as selection variable. All analyses were performed using SPSS for Windows, version 17.0.

## Results

### Prevalence of sexual behaviors

Table 2 displays the prevalence of sexual behaviors in an overall sample in the last one year. A small number of students (10.8%) engaged oral sex and a small number of students (12.6%) had heterosexual intercourse. A few students (2.7%) had same-sex sexual activity, around 46% students had masturbation and more than a half of students (57.4%) viewed pornography movie/video in last one year. In terms to sexual communication, 75.6% students talked about sex with their friends. However, students who talked with parents about sex and contraception accounted for only 13.7% and 7.1%, respectively. In terms of forcing and being forced to have sex, 2.7% students forced a sexual partner to have sex and 1.9% students were forced to have sex in the last one year.

**Table 2:** Prevalence of sexual-related behaviors (n/%)

	**Overall sample**				
**Items**	**Never**	**Less than one monthly**	**Monthly**	**Weekly**	**Daily**
1. Dating (going to dinner, movie, or party with boyfriend/girlfriend).	636(45.3)	397(28.3)	148(10.5)	150(10.7)	73(5.2)
2. Kissing while on a date.	890(63.4)	230(16.4)	98(7.0)	134(9.6)	51(3.6)
3. Petting or fondling (not oral sex).	1012(72.1)	191(13.6)	94(6.7)	77(5.5)	29(2.1)
4. Oral sex (receiving or giving).	1252(89.2)	82(5.8)	39(2.8)	24(1.7)	6(.4)
5. Sexual intercourse with a person of the opposite sex.	1226(87.4)	114(8.1)	40(2.9)	20(1.4)	3(.2)
6. Sexual activity with a person of the same sex	1365(97.3)	25(1.8)	5(.4)	7(.5)	1(.1)
7. Masturbating alone.	757(54.0)	305(21.7)	229(16.3)	103(7.3)	9(.6)
8. Viewing a pornographic movie/video.	598(42.6)	559(39.8)	183(13.0)	54(3.8)	9(.6)
9. Reading pornography magazine.	905(64.5)	409(29.2)	70(5.0)	15(1.1)	4(.3)
10. Talking with your parents about sex.	1211(86.3)	167(11.9)	17(1.2)	8(.6)	0
11. Talking with your parents about contraception.	1304(92.9)	86(6.1)	10(.7)	2(.1)	1(.1)
12. Talking with your boyfriend/girlfriend about sex.	875(62.4)	362(25.8)	98(7.0)	54(3.8)	14(1.0)
13. Talking with your boyfriend/girlfriend about contraception.	1007(71.8)	279(19.9)	70(5.0)	40(2.9)	7(.5)
14. Talking with friends about sex.	343(24.4)	619(44.1)	243(17.3)	143(10.2)	55(3.9)
15. Talking wth friends about contraception.	695(49.5)	485(34.6)	139(9.9)	67(4.8)	17(1.2)
16. Forcing your sexual partner to have sex.	1365(97.3)	22(1.6)	5(.4)	6(.4)	5(.4)
17. Being forced to have sex or having sexually abused.	1376(98.1)	18(1.3)	4(.3)	3(.2)	2(.1)
18. Sexual fantasies.	495(35.3)	505(36.0)	223(15.9)	134(9.6)	46(3.3)

### Gender differences in prevalence of sexual behaviors

There was a varying degree of statistically significant gender differences between male and female group in sexual acts within the last one year. There were strongly significant gender differences in some aspects of sexual behaviors. Males reported more sexual fantasies (84.6%), solitary masturbation (70.3%), and using pornographic videos (86.3%) and magazines (53.6%), talking about sex with friends (85.9%) and sexual fantasies (84.5%) than females (36.1%, 10.9%, 15.6%, 9.3%, 85.9%, and 36%, respectively). There was a moderately significant gender difference in talking with friends about contraception, showing males were more likely to talk with friends about contraception (57.4%) than females (40.4%). Females reported slightly more dating (49.1%), kissing (42.7%) and fondling (29.9%) than males (51.7%, 32.4%, and 26.5%, respectively). It seems males were somewhat more likely to report sexual practices than girls were. And females tended to report more intimacy than boys did (Table 3 and Table 4).

**Table 3:** Prevalence of sexual-related behaviors (n/%) by male/female

	**Male**						**Female**			
**Items**	**Never**	**Less than one monthly**	**Monthly**	**Weekly**	**Daily**	**Never**	**Less than one monthly**	**Monthly**	**Weekly**	**Daily**
1. Dating (going to dinner, movie, or party with boyfriend/girlfriend).	401(48.3)	238(28.6)	95(11.4)	64(7.7)	33(4.0)	234(40.9)	159(27.8)	53(9.3)	86(15.0)	40(7.0)
2. Kissing while on a date.	562(67.6)	121(14.6)	54(6.5)	71(8.5)	23(2.8)	328(57.3)	109(19.1)	44(7.7)	63(11.0)	28(4.9)
3. Petting or fondling (not oral sex).	611(73.5)	113(13.6)	51(6.1)	42(5.1)	14(1.7)	401(70.1)	78(13.6)	43(7.5)	35(6.1)	15(2.6)
4. Oral sex (receiving or giving).	744(89.5)	55(6.6)	19(2.3)	9(1.1)	4(.5)	508(88.8)	27(4.7)	20(3.5)	15(2.6)	2(.3)
5. Sexual intercourse with a person of the opposite sex.	703(84.6)	83(10.0)	27(3.2)	15(1.8)	3(.4)	523(91.4)	31(5.4)	13(2.3)	5(.9)	0
6. Sexual activity with a person of the same sex	803(96.6)	20(2.4)	4(.5)	4(.5)	0	562(98.3)	5(.9)	1(.2)	4(.7)	0
7. Masturbating alone.	247(29.7)	265(31.9)	217(26.1)	95(11.4)	7(.8)	510(89.2)	40(7.0)	12(2.1)	8(1.4)	2(.3)
8. Viewing a pornographic movie/video.	115(13.8)	479(57.6)	174(20.9)	54(6.5)	9(1.1)	483(84.4)	80(14.0)	9(1.6)	0	0
9. Reading pornography magazine.	386(46.5)	360(43.3)	66(7.9)	15(1.8)	4(.5)	519(90.7)	49(8.6)	4(.7)	0	0
10. Talking with your parents about sex.	742(89.3)	74(8.9)	8(1.0)	7(.8)	0	469(82.0)	93(16.3)	9(1.6)	1(.2)	0
11. Talking with your parents about contraception.	780(93.9)	41(4.9)	8(1.0)	2(.2)	0	524(91.6)	45(7.9)	2(.3)	1(.2)	0
12. Talking with your boyfriend/girlfriend about sex.	526(63.3)	188(22.6)	71(8.5)	34(4.1)	12(1.4)	349(61.0)	174(30.4)	27(4.7)	20(3.5)	2(.3)
13. Talking with your boyfriend/girlfriend about contraception.	591(71.1)	154(18.5)	51(6.1)	28(3.4)	7(.8)	416(72.7)	125(21.9)	19(3.3)	12(2.1)	0
14. Talking with friends about sex.	117(14.1)	327(39.4)	203(24.4)	129(15.5)	55(6.6)	226(39.5)	292(51.0)	40(7.0)	14(2.4)	0
15. Talking with friends about contraception.	354(42.6)	285(34.3)	117(14.1)	58(7.0)	17(2.0)	341(59.6)	200(35.0)	22(3.8)	9(1.6)	0
16. Forcing your sexual partner to have sex.	798(96.0)	19(2.3)	5(.6)	4(.5)	5(.6)	567(99.1)	3(.5)	0	2(.3)	0
17. Being forced to have sex or having sexually abused.	811(97.6)	11(1.3)	4(.5)	3(.4)	2(.2)	565(98.8)	7(1.2)	0	0	0
18. Sexual fantasies.	129(15.5)	335(40.3)	197(23.7)	128(15.4)	42(5.1)	366(64.0)	170(29.7)	26(4.5)	6(1.0)	4(.7)

**Table 4:** Sexual-related behaviors: difference by gender (M ± SD)

	**Male**		**Female**		**P value**	***d***
**Items**	**M**	**SD**	**M**	**SD**		
1. Dating (going to dinner, movie, or party with boyfriend/girlfriend).	1.90	1.12	2.19	1.30	.000	**-.26**
2. Kissing while on a date.	1.64	1.10	1.87	1.23	.000	**-.21**
3. Petting or fondling (not oral sex).	1.48	.94	1.58	1.04	.073	**-.11**
4. Oral sex (receiving or giving).	1.16	.55	1.21	.66	.170	-.08
5. Sexual intercourse with a person of the opposite sex.	1.23	.63	1.13	.46	.000	**.20**
6. Sexual activity with a person of the same sex	1.05	.29	1.03	.28	.337	.05
7. Masturbating alone.	2.22	1.02	1.17	.55	.000	**1.36**
8. Viewing a pornographic movie/video.	2.23	.81	1.17	.42	.000	**1.78**
9. Reading pornography magazine.	1.67	.74	1.10	.32	.000	**1.12**
10. Talking with your parents about sex.	1.13	.43	1.20	.45	.006	**-.16**
11. Talking with your parents about contraception.	1.08	.32	1.09	.32	.385	-.05
12. Talking with your boyfriend/girlfriend about sex.	1.58	.91	1.52	.77	.184	.07
13. Talking with your boyfriend/girlfriend about contraception.	1.44	.82	1.35	.65	.016	**.13**
14. Talking with friends about sex.	2.61	1.11	1.72	.70	.000	**.99**
15. Talking with friends about contraception.	1.92	1.01	1.47	.65	.000	**.53**
16. Forcing your sexual partner to have sex.	1.07	.43	1.02	.19	.001	**.20**
17. Being forced to have sex or having sexually abused.	1.04	.32	1.01	.11	.010	**.16**
18. Sexual fantasies.	2.54	1.08	1.45	.70	.000	**1.23**

### Factors correlated with sexual behaviors by males/females

Linear regression analyses were performed to examine factors (age, grade, discipline, education aspiration, romantic relationship, sex education experience, rural/urban area and time spent online) linked to sexual related behaviors for male and female students, respectively. The analysis found that five factors for males were significantly associated with sexual behaviors in last one year: romantic relationship (*β* <−.29, *p* < 0.001), received sex education (*β* < −.13, *p* < 0.001), education aspiration (*β* < −.09, *p* < 0.05), time spent online (*β <* .09, *p* < 0.01) and area (*β < −*.07*, p* < 0.05). The five factors could explain 19% of males’ sexual behaviors. Two factors for females were significantly correlated with sexual behaviors: romantic relationship (*β* < −.46, *p* < 0.001) and area (*β < −*.09, *p* < 0.01). The two factors could explain 27% of females’ sexual behaviors. Age, grade and discipline were not significantly associated with sexual behaviors in both male and female group (Table 5).

**Table 5:** Predictors of sexual-related behaviors: differences by gender

		**Male**					**Female**				
**DV**	**IV**	***B***	**SE**	***β***	***t***	***ΔR***^***2***^	***B***	**SE**	***β***	***t***	***ΔR***^***2***^
Sexual behaviors	Age	.01	.03	.02	.42	.19	.01	.02	.01	.360	.27
	Grade	.40	.32	.05	1.07		.44	.28	.07	1.60	
	Discipline	.51	.59	.03	.86		-.47	.47	-.04	.99	
	Education aspiration	-1.00	.39	-.09	-2.56*		-.53	.39	-.05	-1.35	
	Relationship experience	-4.70	.52	-.29	-9.02***		-5.91	.49	-.46	-11.98***	
	Sex education experience	-3.06	.79	-.13	-3.88***		-.76	.84	-.03	-.90	
	Area	-1.27	.57	-.07	-2.22*		-1.13	.48	-.09	-2.34*	
	Time spent online	.87	.31	.09	2.81**		-.41	.34	-.04	-1.19	

Age: 18~19 year-old were coded as “1”, 20~21 years old were coded as “2”, and 22~25 years old were coded as “3”.

Grade: Year1 was code as “1”, Year 2 was codes as “2”, Year 3 was coded as “3”, and Year 4 was coded as “4”.

Discipline: Arts was coded as “0” and Science was coded as “1”.

Education aspiration: Bachelor degree was coded as “1”, Master degree was coded as “2”, and PhD degree was coded as “3”.

Relationship experience: Having relationship experience was coded as “0” and no relationship experience was coded as “1”.

Sex education experience: Having sex education experience was coded as “0” and no sex education experience was coded as “1”.

Area: urban area was coded as “0” and rural area was coded as “1”.

Time spent online: Almost no was coded as “1”, ≤ 2hours was coded as “2”, 2~4 hours was coded as “3”, and ≥ 4hours was coded as “4”.

## Discussion

The present study found that rates of heterosexual intercourse reported by the university students in Hefei who responded to our study were 12.6% (15.4% males and 8.5% females). These rates fall within the ranges reported by Chinese university students in other Chinese cities since 1995 [16,28,29]. During the last decade, the rates of heterosexual intercourse among Chinese university students do not appear to have undergone a dramatic change, remaining the similar with or no big different from rates observed in neighboring regions or countries. For example, it was reported that 22% of never married youth aged 20 years having had sex in Taiwan in 2004 [30]. And in the Survey Assessment of Vietnamese Youth conducted in late 2003, it was found that 16.7% male and 2.4% female aged 18 to 25 years engaged sexual intercourse [31]. It may be because the regions in Asia, such as Taiwan, Korea, Vietnam and Japan, share the same Confucian-based traditional culture expecting in terms of sexuality that men and women should conduct themselves properly from an emotional distance at all times and not have any contact before marriage [32]. Although they have been open to outside influences socially, culturally and economically for different periods and in different ways, their sharing traditional culture still rooted in societies deeply. Compared to western countries, the percentage in China remained much lower than rates observed in the USA that 80% of male university students and 73% of female university students had heterosexual intercourse and in Scotland that around 74% university students had heterosexual intercourse during the 1990s and early 2000s [33,34]. This may be related to vast differences in cultural and social context. A specific example is that the Chinese Ministry of Education prohibited marriage among university students until 2005 and the universities offer a context which discouraged university students’ sexual activities. In China, many universities have direct and indirect regulations that limit intimate relationships between students of opposite sex in school. For example, every student must live in school and males were not allowed to enter female dormitories; students must come back to the dormitory before 10:30 pm since the gates of dormitories usually close at 10:30 pm, with the lights turned off at 11:30 pm. In addition to heterosexual intercourse, our results also clearly indicate that there were other acts among university students, with both male and female students having oral sex, the same-sex activities, and forcing and being forced to have sex. The results may indicate that sex education not only advocates abstinence as a good way for safe sex, but also provides comprehensive sex education including sexual knowledge about reproductive health, condom and contraception use, proper and responsible sexual attitude for protective, and safe sex behaviors among young people.

There are six items in the sexual behaviors questionnaire asking students about their communication with other people on sexual topics or other ways of acquiring sexual knowledge (e.g., viewing pornography videos or magazines). With respect to communication on sexual topics, the results of the current study suggest that parent-adolescent communication on sex was quite infrequent in China than in Western countries. A study conducted in Sweden reported that 40% of male and 60% of female high school students had talked with their parents about sex [35]. However, in the current study, only 13.7% (10.7% males and 18% females) students talked with parents about sex, and only 7.1% (6.1% males and 8.4% female) students talked with parents about contraception in the last one year in China. Given the important role parents play in adolescents’ life [35], parental involvement in adolescent sex education needs to be enhanced. For example, parents should be encouraged to communicate with and educate children about sex in an environment of openness when they are most drawn to sexual behaviors during their adolescence. The study also revealed the large proportion of students, especially male students, who saw pornography such as books/magazines/videos/websites. It may suggest that pornography may be a ready source of basic information about sex for Chinese youth and may have had some influence on respondents’ sexual practices. It becomes quite necessary to incorporate pornography topics in sex education for university students in China [15]. For example, educating youth about the realism of pornography and the relationship between media and life; encouraging students to reflectively think and discuss what the possible benefits and harmful effects of pornography are for young people, why people use it, and what the law says about it.

This study found significant gender differences (male > female) in prevalence of heterosexual intercourse, masturbation, sex fantasies, exposure to pornographic media; these differences were consistent with previous studies done in China and USA [29,36,37]. The difference of heterosexual intercourse may be explained by noting that whereas premarital heterosexual intercourse for boys is considered a socially acceptable rite of passage, girls tend to be labeled and stigmatized and are often blamed for sexual encounters that could result in pregnancy and sexually transmitted infections [38]. Attitudes and beliefs from family and society still expected men to take responsibility for initiating and ending sexual activity. Women are expected to be virgins before marriage and less sexually initiating than men [14,39]. Gender differences in sex fantasy, masturbation and pornography use may also be partially due to sex drive. Research suggests that men on average have a stronger sex drive and are more aroused by pornography than women do [40]. Alternatively, the large gender differences in sex fantasy, masturbation and pornography use may be explained by socially desirable responding. Stigma continues to be associated with female autoerotic behaviors particularly in different Chinese communities; therefore, women may underreport rates of masturbation or pornography use [38].

Consistent with previous studies, it was found that the incidence of sexual behaviors in the past year for university students were positively related to having romantic relationship experience, received sex education, lower education aspiration, longer time spent online and living urban area for male and having romantic relationship experience and living in urban area for female.

Among all the factors identified predicting university students’ sexual behaviors, having romantic relationship experience had the strongest explanative power for both male and female. Several studies have evidenced that dating, especially steady romantic relationship is a prominent factor associated with sexual behaviors. Having a boyfriend or girlfriend may increase the opportunity for engaging in intimate and precoital behaviors, such as kissing and fondling, which may be followed by sex. Furthermore, having a boyfriend or girlfriend may expose a youth to a new set of friends, who may share more permissive norms about sex; many studies have demonstrated that youth whose peer norms encourage sexual activity have an increased likelihood of being sexually active. Thus, young people in romantic relationships have extra need for information about intimacy and sexual risk and safety. The study suggests the importance of targeting education efforts toward young people in romantic relationship [41]. Schools and parents should help young people, in particular those in romantic relationship, to develop skills and abilities in relationship and intimacy including teaching sexual health knowledge, advocating safe sexual behaviors, and to make rational sexual decision.

Received sex education has the second most influential source to explain male students’ sexual behaviors. For males, students with received sex education showed significantly more sexual behaviors than those without such experiences. This seems to be inconsistent with the “good intention” of Chinese mainstream society that sex education should delay sexual initiation and decrease sexual activities among adolescents and young adults [42]. However, it seems that recent data have shown that the “good intention” did not protect young people better [43-45]. On the other hand, sex education subjects are elective courses in Chinese universities. It is also possible that students who are interested in sexuality or have sexual experiences are more likely to choose the related subjects. Relationship between sex education and adolescents’ sexual behaviors is complex [46]. What role does sex education play? The researchers agree with Pan’s point of view: “Sex education neither alone plays ‘fire extinguisher’ role, not functions as ‘accelerant’; the ultimate goal of sex education is to help all individuals, especially the next generation, enjoy a ‘happy sex life’ as much as possible [46].” Namely, sex education should assist young people in developing a positive view of sexuality, provide them with information they need to take care of their sexual health, and help them acquire skills to make decisions now and in the future. However, considering China’s lack of an open and free social climate which normally plays a very important role in promoting sex education, it is very important to use the university system to introduce sexuality. Firstly, the university system appears to be a “safe” place or platform to incorporate debates and understandings of sexualities. Universities also have much more freedom to talk about sexuality than other places; therefore, debates can be deeper and more analytical. Furthermore, university students tend to be more open and to more easily adopt new ideas and perspectives on sexualities [47].

Another factor correlated with sexual behaviors was educational aspiration for males. We found that education aspiration could negatively predicted male students’ sexual behaviors, namely the higher education aspiration, the less sexually active. The finding further confirmed prior studies that commitment to doing well in academic protected respondents from becoming sexually active and more sexual partners [48]. Finally, time spent online was the last factor correlated with sexual behaviors for males. Our study found time spent online could slightly predict male students’ sexual behaviors, namely the longer surfing internet time, the more sexually active. But it couldn’t predict female students’ sexual behaviors. This may be because male students reported much higher rates of visiting pornographic websites, partner seeking and engaging online risky behaviors, which was closely associated with sexual behaviors including risky sexual behaviors [49]. Appropriate guidance on their Internet use will be needed in China regarding the possible future impact of the Internet and pornographic media on the sexual behaviors of young people. For example, carefully monitoring and reducing online sexual risk behaviors among youth, especially males, and properly utilizing Internet as an informative source for sex education.

This study had several limitations. First, its cross-sectional design prevented us from identifying cause-and-effect associations, such as whether education aspiration decreased the prevalence of male students’ sexual behaviors could not be determined in this study. Second, the results obtained in this study should not be generalized to all Chinese young people or to all Chinese university students, since our sample was limited to university students within one capital city and socio-demographic characteristics are greatly diverse within Chinese provinces. Finally, the possible bias introduced by under-reporting should be noted. Measurements of sexual activity in this study were based on self-reports and participant sensitivity, especially female students, regarding sexual behavior may have led to under-reporting because of social desirability effect. Social desirability scale may be included in future survey.

## Conclusion

Our results revealed that the frequency of sexual activities among university students in Hefei varied with varying degree of gender differences, such as masturbation, seeing pornography, heterosexual intercourse and sexual communication. Moreover, our results showed that sexual behaviors were significantly predicted by romantic relationship, received sex education, education aspiration, time spent online and area for male students, and romantic relationship and area for female students. This information is important for policy makers and sex educators to develop effective and feasible strategies targeting at promoting sex education for Chinese university students.

## Competing interests

The authors declare that they have no competing interests.

## Authors’ contributions

All authors contributed to the design of this research. XC and LY performed the statistical analysis and drafted the manuscript; XC was supervised by SW in the study idea and survey and SW also supervised the research, statistical analysis and revised the manuscript. All authors read and approved the final manuscript.

## Pre-publication history

The pre-publication history for this paper can be accessed here:

http://www.biomedcentral.com/1471-2458/12/972/prepub

## References

[B1] Crossette B Reproductive Health and the Millennium Development Goals: The Missing Link Stud Fam Plann 2005 36 1 71 79 10.1111/j.1728-4465.2005.00042.x 15828526

[B2] Marston C King E Factors that shape young people’s sexual behaviour: a systematic review Lancet 2006 368 9547 1581 1586 10.1016/S0140-6736(06)69662-1 17084758

[B3] Tang J Gao XH Yu YZ Ahmed NI Zhu HP Wang JJ Du YK Sexual Knowledge, attitudes and behaviors among unmarried migrant female workers in China: a comparative analysis BMC Publ Health 2011 11 917 10.1186/1471-2458-11-917 PMC3259080 22151661

[B4] Wellings K Collumbien M Slaymaker E Singh S Sexual and Reproductive Health 2: Sexual behaviour in context: a global perspective Lancet 2006 368 9548 1706 1728 10.1016/S0140-6736(06)69479-8 17098090

[B5] Reinisch JM Hill CA Sanders SA Ziemba-Davis M High-Risk Sexual Behavior at a Midwestern University: A Confirmatory Survey Fam Plann Perspect 1995 27 2 79 82 10.2307/2135910 7796901

[B6] Gökengin D Yamazhan T Özkaya D Aytuǧ S Ertem E Arda B Serter D Sexual Knowledge, Attitudes, and Risk Behaviors of Students in Turkey J Sch Health 2003 73 7 258 263 10.1111/j.1746-1561.2003.tb06575.x 14513628

[B7] Eisenberg M Differences in sexual risk behaviors between college students with same-sex and oppositve-sex experience: Results from a national survey Arch Sex Behav 2001 30 6 575 589 10.1023/A:1011958816438 11725456

[B8] Fiebert M Osburn K Effect of gender and ethnicity on self reports of mild, moderate and severe sexual coercion Sexuality & Culture 2001 5 2 3 11 10.1007/s12119-001-1015-2

[B9] Kaestle C Allen K The Role of Masturbation in Healthy Sexual Development: Perceptions of Young Adults Arch Sex Behav 2011 40 5 983 994 10.1007/s10508-010-9722-0 21293916

[B10] Gert MH Gender Differences in Pornography Consumption among Young Heterosexual Danish Adults Arch Sex Behav 2006 35 5 577 585 10.1007/s10508-006-9064-0 17039402

[B11] Zhang SB An investigation on college students about AIDS knowledge AIDS Bull 1993 4 78 81

[B12] Li H Zhang KL The progress of social behavior science related to HIV/AIDS Chin J Prev Med (in Chinese) 1998 2 120 124

[B13] Research Group on Sex Education among University Students Report on sexual behavior survey among Chinese university students in 2000 Youth Study (in Chinese) 2001 12 31 39

[B14] Pan SM Sexual value and sexual behavior among college students in contemporary China 2008 Retrieved from http://blog.sina.com.cn/s/blog_4dd47e5a0100ap9l.html

[B15] Ma QQ Kihara MO Cong LM Xu GZ Zamani S Ravari SM Kihara M Sexual behavior and awareness of Chinese university students in transition with implied risk of sexually transmitted diseases and HIV infection: A cross-sectional study BMC Publ Health 2006 6 232 10.1186/1471-2458-6-232 PMC1586016 16981985

[B16] Zuo XY Lou CH Gao E Cheng Y Niu HF Zabin LS Gender Differences in Adolescent Premarital Sexual Permissiveness in Three Asian Cities J Adolesc Health 2012 50 S18 S25 22340852 10.1016/j.jadohealth.2011.12.001 PMC4235609

[B17] Wu J Xiong G Shi S Study on sexual knowledge, attitudes and behaviors of adolescents Chinese Journal of Child Health Care (in Chinese) 2007 15 2 120 121

[B18] Wang B Hertog S Meier A Lou C Gao E The Potential of Comprehensive Sex Education in China: Findings from Suburban Shanghai Int Fam Plan Perspect 2005 31 2 63 72 10.1363/3106305 15982947

[B19] Bastien S Kajula L Muhwezi W A review of studies of parent–child communication about sexuality and HIV/AIDS in sub-Saharan Africa Reprod Heal 2011 8 1 25 10.1186/1742-4755-8-25 PMC3192730 21943095

[B20] Marín BV Kirby DB Hudes ES Coyle KK Gómez CA Boyfriends, girlfriends and teenagers’ risk of sexual involvement Perspect Sex Reprod Health 2006 38 2 76 83 10.1363/3807606 16772188

[B21] Strasburger VC Wilson BJ Jordan AB Children, adolescents, and the media 2009 2 Thousand Oaks, CA: Sage

[B22] Li SH Huang H Cai Y Xu G Huang FR Shen XM Characteristics and determinants of sexual behavior among adolescents of migrant workers in Shangai (China) BMC Publ Health 2009 9 195 10.1186/1471-2458-9-195 PMC2706248 19538756

[B23] Ma QQ Kihara MO Cong LM Early initiation of sexual activity: a risk factor for sexually transmitted diseases, HIV infection, and unwanted pregnancy among university students in China BMC Publ Health 2009 9 111 10.1186/1471-2458-9-111 PMC2674603 19383171

[B24] Zhang HM Liu BH Zhang GZ Risk Factors for Sexual Intercourse among Undergraduates in Beijing Chin J Sch Health (in Chinese) 2007 28 12 1057 1059

[B25] Song SQ Zhang Y Zhou J Comparison on sex knowledge, attitude, behavior, and demand between common high school students and occupational high school students Maternal and Child Health Care of China 2006 21 4 507 509

[B26] Lief HI Fullard W Devlin SJ A New Measure of Adolescent Sexuality: SKAT-A Journal of Sex Education and Therapy 1990 16 2 79 91

[B27] Fisher TD Davis CM Yarber WL Davis SL Handbook of Sexuality-Related Measures 2010 New York: Routledge

[B28] Li A Wang A Xu B University students’ attitudes toward premarital sex and their sexual activity in Beijing Sexology (in Chinese) 1998 7 19 24

[B29] Zhang LY Gao X Dong ZW Tan YP Wu ZL Premarital Sexual Activities Among Students in a University in Beijing, China Sex Transm Dis 2002 29 4 212 215 10.1097/00007435-200204000-00005 11912462

[B30] Chiao C Yi CC Adolescent premarital sex and health outcomes among Taiwanese youth: Perception of best friends’ sexual behavior and the contextual effect AIDS Care 2011 23 1083 1092 10.1080/09540121.2011.555737 21562995

[B31] de Lind van Wijngaarden JW Exploring factors and processes leading to HIV risk among the most vulnerable children and adolescents in Vietnam (literature review 2006 Hanoi, Vietnam: UNICEF

[B32] Hong W Yamamoto J Chang DS Sex in Confucian society J Am Acad Psychoanal 1993 21 405 419 8226181 10.1521/jaap.1.1993.21.3.405

[B33] Reinisch JM Hill CA Sanders SA Ziemba-Davis M High-risk sexual behavior at Midwestern university: A confirmatory survey Fam Plan Perspect 1995 27 79 82 10.2307/2135910 7796901

[B34] Raab GM Burns SM Scott G Cudmore S Ross A Gore SM O’Brien F Shaw T HIV prevalence and risk factors in university students AIDS 1995 9 191 197 7718191

[B35] Zhang LY Li XM Shah IH Baldwin W Stanton B Parent–adolescent sex communication in China Eur J Contracept Reprod Health Care 2007 12 2 138 147 10.1080/13625180701300293 17559012

[B36] Gao Y Lu ZZ Shi R Sun XY Cai Y AIDS and sex education for young people in China Reprop Fertil Dev 2001 13 729 737 10.1071/RD01082 11999327

[B37] Petersen JL Hyde JS Gender Differences in Sexual Attitudes and Behaviors: A Review of Meta-Analytic Results and Large Datasets J Sex Res 2001 48 2–3 149 165 21409712 10.1080/00224499.2011.551851

[B38] Kaljee LM Green M Riel R Sexual stigma, sexual behaviors, and abstinence among Vietnamese adolescents: Implications for risk and protective behaviors for HIV, sexually transmitted infections, and unwanted pregnancy J Assoc Nurs AIDS Care 2007 18 48 59 10.1016/j.jana.2007.01.003 PMC2063998 17403496

[B39] Baumeister RF Catanese KR Vohs KD Is there a gender difference in strength of sex drive? Theoretical views, conceptual distinctions, and a review of relevant evidence Personality and Social Psychology Review 2001 5 242 273 10.1207/S15327957PSPR0503_5

[B40] Wang B Li XM Bonita S Sexual attitudes, pattern of communication, and sexual behavior among unmarried out-of-school youth in China BMC Publ Health 2007 7 189 10.1186/1471-2458-7-189 PMC1965477 17672903

[B41] VanOss Marín DB Kirby B Hudes ES Coyle KK Gómez CA Gómez: Boyfriends, Girlfriends and Teenagers’ Risk of Sexual Involvement Perspect Sex Reprod Health 2006 38 2 76 83 10.1363/3807606 16772188

[B42] Dawson DA Effects of Sex Education on Adolescent Behavior Fam Plann Perspect 1986 18 4 162 170 10.2307/2135325 3792529

[B43] Xu Q Tang SL Pau G Unintended pregnancy and induced abortion among unmarried women in China: a systematic review BMC Health Service Research 2004 4 1 4 10.1186/1472-6963-4-1 PMC333425 14736336

[B44] China Ministry of Health China statistical summary for health for 2003, 2004, 2005 http://www.moh.gov.cn/news/sub_index.aspx?tp_class=C3

[B45] UNAIDS UNAIDS report on the global AIDS epidemic 2010 Retrieved from http://www.unaids.org/globalreport/global_report.htm

[B46] Pan SM Talking about Adolescents’ sex education Population Research 2002 26 6 20 28

[B47] Huang YY Pan SM Peng T Gao YN Teaching Sexualities at Chinese Universities: Context, Experience, and Challenges International Journal of Sexual Health 2009 21 4 282 295 10.1080/19317610903307696

[B48] Roberts SR Moss RL The Impact Family Structure has on Sexual Activity and Educational Aspirations for African American adolescents ages 12–17 Proceedings of the 3rd Annual GRASP Symposium 2007 Wichita State University 155 156

[B49] Hong Y Li XM Mao R Stanton B Internet Use among Chinese College Students: Implications for Sex Education and HIV Prevention Cyberpsychol Behav 2007 10 11 161 169 17474831 10.1089/cpb.2006.9973

